# An Insulin-to-Insulin Regulatory Network Orchestrates Phenotypic Specificity in Development and Physiology

**DOI:** 10.1371/journal.pgen.1004225

**Published:** 2014-03-27

**Authors:** Diana Andrea Fernandes de Abreu, Antonio Caballero, Pascal Fardel, Nicholas Stroustrup, Zhunan Chen, KyungHwa Lee, William D. Keyes, Zachary M. Nash, Isaac F. López-Moyado, Federico Vaggi, Astrid Cornils, Martin Regenass, Anca Neagu, Ivan Ostojic, Chang Liu, Yongmin Cho, Deniz Sifoglu, Yu Shen, Walter Fontana, Hang Lu, Attila Csikasz-Nagy, Coleen T. Murphy, Adam Antebi, Eric Blanc, Javier Apfeld, Yun Zhang, Joy Alcedo, QueeLim Ch'ng

**Affiliations:** 1 MRC Centre for Developmental Neurobiology, King's College London, London, United Kingdom; 2 Friedrich Miescher Institute for Biomedical Research, Basel, Switzerland; 3 Biozentrum, University of Basel, Basel, Basel, Switzerland; 4 Dept of Systems Biology, Harvard Medical School, Boston, Massachusetts, United States of America; 5 Dept of Organismic and Evolutionary Biology, The Center for Brain Science, Harvard University, Cambridge, Massachusetts, United States of America; 6 Lewis-Sigler Institute for Integrative Genomics and Dept of Molecular Biology, Princeton University, Princeton, New Jersey, United States of America; 7 Research and Innovation Center, Fondazione Edmund Mach, San Michele all'Adige, Italy; 8 School of Chemical and Biomolecular Engineering, Georgia Institute of Technology, Atlanta, Georgia, United States of America; 9 Dept of Biological Sciences, Wayne State University, Detroit, Michigan, United States of America; 10 Institute for Mathematical and Molecular Biomedicine, King's College London, London, United Kingdom; 11 Randall Division of Cell and Molecular Biophysics, King's College London, London, United Kingdom; 12 Max Planck Institute for Biology of Ageing, Koeln, Germany; Stanford University Medical Center, United States of America

## Abstract

Insulin-like peptides (ILPs) play highly conserved roles in development and physiology. Most animal genomes encode multiple ILPs. Here we identify mechanisms for how the forty *Caenorhabditis elegans* ILPs coordinate diverse processes, including development, reproduction, longevity and several specific stress responses. Our systematic studies identify an ILP-based combinatorial code for these phenotypes characterized by substantial functional specificity and diversity rather than global redundancy. Notably, we show that ILPs regulate each other transcriptionally, uncovering an ILP-to-ILP regulatory network that underlies the combinatorial phenotypic coding by the ILP family. Extensive analyses of genetic interactions among ILPs reveal how their signals are integrated. A combined analysis of these functional and regulatory ILP interactions identifies local genetic circuits that act in parallel and interact by crosstalk, feedback and compensation. This organization provides emergent mechanisms for phenotypic specificity and graded regulation for the combinatorial phenotypic coding we observe. Our findings also provide insights into how large hormonal networks regulate diverse traits.

## Author Summary

Insulin signaling is widely implicated in regulating diverse physiological processes ranging from metabolism to longevity across many animal species. Many animals have multiple insulin-like peptides that can regulate the activity of this signaling pathway. For example, while humans have ten, including the well-studied insulin hormone, the nematode *Caenorhabditis elegans* has forty such peptides. The similarity among these insulin-like peptides led to the predominant notion that widespread redundancy occurs among these peptides. Contrary to this notion, we find that the forty insulin-like peptides in the nematode *C. elegans* have specific and distinct effects on eight different physiological outputs that range from development, stress responses, lifespan and reproduction. Interestingly, we also find that these peptides regulate each other at the transcriptional level to form a signaling network. In addition, we observe that this network is organized into parallel circuits, whose activities are affected by compensation, feedback and crosstalk. Finally, the organization of the network helps to explain how different combinations of peptides generate specific outputs and captures the complexity of how these peptides orchestrate an animal's physiology through distinct peptide-to-peptide signaling circuits.

## Introduction

The organization and integration of multiple signals endow intercellular regulatory networks with information processing capabilities. For example, hormones modulate physiology and maintain homeostasis in variable environments [reviewed in 1], and morphogens give rise to intricate patterns during development [reviewed in 2]. Nevertheless, how simple circuits are organized into complex networks that perform sophisticated functions is not fully understood.

The ILPs are a superfamily of hormones that regulate many processes, including development, cell proliferation, energy metabolism, neuronal function, reproduction stress resistance, and longevity [3]–[15]. Canonical ILP signaling is mediated by a receptor tyrosine kinase pathway that culminates in the regulation of FOXO transcription factors and other regulatory molecules [16]. In *Caenorhabditis elegans*, this occurs via the DAF-2 ILP receptor tyrosine kinase, which signals through DAF-16 FOXO [6], [17]–[19]. The importance of ILP signaling is underscored by the conservation of both the signal transduction pathway and the processes they regulate. Indeed, a *C. elegans* ILP (INS-6) resembles human insulin structurally and can bind and activate the human insulin receptor [20].

Most animal genomes encode multiple ILPs: humans have 10 [21]; *Drosophila melanogaster* has 8 [22]–[24]; and *C. elegan*s has 40 [25]–[27]. Small-scale studies have shown that certain ILPs can regulate other ILPs [4], [24], [28]–[31], and that ILPs can act as either agonists or antagonists of their receptor to differentially affect multiple processes [5], [24], [27]. How do such simple interactions between these hormones generate complex functionality? Here, we address this question by an integrated analysis of the *C. elegans* ILPs during larval development, stress resistance, reproduction and lifespan. We systematically tested the function of *C. elegans* ILPs in the control of diverse phenotypes. In contrast to the common notion of broad redundancy among ILPs [32], we now provide evidence supporting a combinatorial code of action that maps the ILPs to multiple phenotypes. We also uncover the existence of a *C. elegans* ILP-to-ILP regulatory network that reveals the mechanisms through which multiple functionally diversified ILPs interact to regulate complex developmental and physiological traits. Thus, our analysis of the ILP-to-ILP network provides organizational principles for multiple-gene families and signaling networks.

## Results

### An ILP Combinatorial Code for Coordinating Development and Physiology

As in many animals, the *C. elegans daf-2* insulin/ILP receptor pathway affects multiple physiological processes, including development, aging, pathogen resistance, thermotolerance and reproduction [6], [8], [9], [17], [33]–[37]. The *C. elegans* ILP pathway also regulates entry into a specialized form of larval arrest known as dauer that forms preferentially under adverse conditions, such as high temperature, high population density, and low dietary sterols and food levels [reviewed in 6]. Under favorable conditions, animals exit the dauer stage to resume reproductive growth. Dauer exit is also regulated by the ILP pathway [34], [38], which suggests that ILPs function to regulate developmental plasticity in response to complex environmental cues [5], [31].

**Figure 1:**
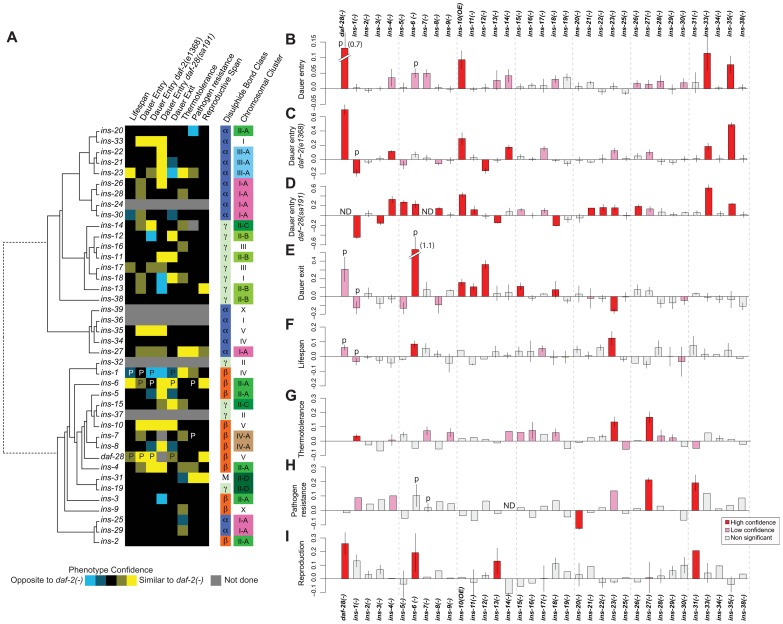
Phenotypes of ILP mutants. (**A**) Heat map summarizing the confidence and direction of each phenotype for 35 ILP mutants (indicated in the legend, bottom left). “P” labels previously published results that we analyzed together with this dataset [66]. Phenotypes were aligned to a tree of the 40 *C. elegans* ILPs based on protein sequence similarity (left); structural class (Pierce et al., 2001) and chromosomal organization (right). (**B**–**I**) indicate the magnitude of each phenotype compared to same-trial controls. (**B**) dauer entry; (**C**) dauer entry in the *daf-2(e1368)* background; (**D**) dauer entry in the *daf-28(sa191)* background; (**E**) dauer exit in the *daf-2(e1368)* background; (**F**) lifespan; (**G**) thermotolerance; (**H**) pathogen resistance; and (**I**) reproductive span. Bars in (**B–I**) were colored as indicated in the legend (bottom right). Y-axes in (**B–D**) indicate percentage of dauers normalized to same trial controls. Y-axes in (**E–I**) indicate mean durations of dauer exit (**E**), lifespan (**F**), survival at 34.5°C (**G**), survival on PA14 (**H**), and reproductive span (**I**), normalized to same-trial controls.

Previous studies, which focused on a few ILPs, suggest that different phenotypes are modulated by distinct ILPs [4], [5], [11], [26], [27]. These ILPs can exhibit complex functional interactions in the regulation of certain phenotypes [4], [5]. Together, these observations have raised the possible existence of an ILP combinatorial code in regulating physiology, in contrast to the prevailing notion of widespread redundancy as a feature of the ILPs and other gene families [32]. We tested this possibility by mapping the relationships between the 40 *C. elegans* ILPs, *ins-1* to *ins-39* and *daf-28* [25]–[27], and their developmental and physiological outputs. We systematically tested mutants in 35 ILPs for 8 distinct developmental and physiological phenotypes (Figures 1 and S1). Thirty-four of these mutations delete part or all of the coding sequence of an ILP and are predicted to be null mutations. One mutation, *ins-10(tm3498)*, contained a deletion in the genomic sequence and a duplication that overexpressed the intact coding sequence, which represents a gain-of-function allele (Table S5, Figure S2 and Materials and Methods). To minimize genetic background effects, all mutants were outcrossed 6 times to wild type.

We used well-established procedures to score the ILP mutants and applied several statistical criteria to classify the phenotypes as high or low confidence based on statistical significance and reproducibility (see Materials and Methods). We also confirmed the roles of many ILPs that showed new phenotypes or represented key conclusions in this study by rescuing their phenotypes with a transgene bearing the wild-type copy of the corresponding gene, as described in the following sections and in Table S3. We included our previously published work in the analysis for comparison (Figures 1 and S1, Table S2) [4], [5]. Importantly, we implicated distinct combinations of ILPs in every process tested and ascribed new functions to more than half of the *C. elegans* ILPs (Figure 1): 66% (23/35) of those tested showed at least one high-confidence phenotype, and 89% (31/35) showed high- or low-confidence phenotypes. We focused our analysis on the high-confidence hits.

#### Dauer entry

First, we screened single mutants at 27°C. In addition to the previously identified *daf-28* [5], we found increased dauer entry rates in the loss-of-function alleles of *ins-33* (∼11% increase) and *ins-35* (∼8% increase), and the gain-of-function allele of *ins-10* (∼9% increase) (Figure 1B, Table S2). Although this screen yielded high-confidence hits, we performed additional sensitized screens for two reasons. Besides the possibility of redundancy, the low dauer entry rate in wild-type animals made it difficult to detect ILPs that act to promote dauer entry. Thus, we screened for enhancers and suppressors under two conditions where intermediate levels of dauer entry were observed: in mutants with the weak *daf-2(e1368)* mutation at 22.5°C [34] (Figure 1C, Table S2), or the dominant negative *daf-28(sa191)* mutation at 20°C [26] (Figure 1D, Table S2). Screening in the *daf-2(e1368)* background identified a new high-confidence suppressor: *ins-12* (∼16% less dauers); and enhancers of dauer entry: *ins-4* (∼12%), *ins-14* (∼17%), *ins-33* (∼18%) and *ins-35* (∼49%). Screening in the dominant negative *daf-28(sa191)* background identified additional high-confidence enhancers with increases in dauer entry rates: *ins-5* (27%), *ins-8* (14%), *ins-11* (∼12%), *ins-21*(∼15%), *ins-22* (16%), *ins-23* (16%) and *ins-26* (∼19%); and suppressors with decreases in dauer entry rates: *ins-3* (∼15%) and *ins-13* (∼15%), along with previously reported ILPs [5], [27], [39]. We rescued the dauer entry phenotypes of *ins-12*, *ins-14*, *ins-33* and *ins-35* in the *daf-2(e1368)* background; and of *ins-3*, *ins-4*, *ins-5*, *ins-21* and *ins-26* in the *daf-28(sa191)* background (Table S3).

#### Dauer exit

We tested the ILP mutants for the ability to modulate the rate of dauer exit in the *daf-2(e1368)* background, which at 25°C, drives all animals into dauers that exit over several days (Figure 1E, Table S2) [5], [34]. Besides previously identified ILPs [5], [40], delayed dauer exit was observed in strains with deletions in *ins-11* (∼11%), *ins-12* (∼36%), *ins-15* (∼11%) and *ins-18* (8%), and the *ins-10* gain-of-function allele (16%). Premature dauer exit was observed in strains with the *ins-23* deletion (∼16% less time in the dauer state). We observed rescue of the dauer exit phenotypes for *ins-12* and *ins-15* (Table S3).

#### Lifespan

We screened for lifespan phenotypes at 20°C, except for *ins-1* mutants that were only tested previously at 25°C [5]. We identified *ins-23(−)* as a new ILP mutant with a high-confidence long-lived phenotype (∼12% increase) (Figure 1F, Table S2) which was rescued by an *ins-23(+)* transgene (Table S3). *ins-6* mutants were reported to be slightly long-lived at 25°C [5]; our results indicate that *ins-6* also increased lifespan by ∼8% at 20°C. We found no mutation that reduced lifespan significantly.

#### Thermotolerance

Using an automated system that quantifies motion [41], we measured the survival of ILP mutants at 34.5°C, a temperature that kills *C. elegans*. We found that *ins-27* mutants showed the most significant increase in thermotolerance (∼17%); smaller increases were found in *ins-23* (∼13%) and *ins-1* mutants (∼4%) (Figure 1G, Table S2). We rescued the thermotolerance phenotype of *ins-27* (Table S3).

#### Pathogen resistance

We quantified survival on a clinical isolate (PA14) of *Pseudomonas aeruginosa* [42] (Figure 1H, Table S2) and found increased resistance to this pathogen at 25°C for *ins-27* (∼19%) and *ins-31* (∼17%) mutants, for which we also observed rescue (Table S3). Conversely, *ins-20* mutants were significantly more susceptible to PA14 (∼11%).

#### Reproductive longevity

By measuring the reproductive period at 15°C (Figure 1I, Table S2), we found longer reproductive periods than wild-type controls for *daf-28* (∼26%), *ins-6* (∼19%), *ins-13* (∼13%) and *ins-31* (∼21%) deletion mutants. We rescued the reproductive longevity phenotype of the *ins-31* deletion mutant (Table S3).

Some ILPs are organized into gene clusters, while others are isolated [27]. Structurally, the ILPs are grouped based on the predicted disulfide bond pattern (α, β, and γ) or insulin-like repeats unique to *ins-31* [27]. We compared gene clustering and ILP function against a phylogeny tree of all 40 ILPs based on protein sequence and found that gene function and clustering did not correlate with protein sequence similarity (Figure 1A). Furthermore, neither gene clustering nor structural classification strongly predicts phenotype. Instead, our data suggest functional divergence after local tandem duplication, which can occur rapidly, such as in the *ins-2* to *ins-6* cluster where *ins-6* and *ins-3* had opposite effects on dauer entry in the *daf-28(sa191)* background, and *ins-2* had no effect (Figure 1). We also did not detect any significant correlation between expression and function when we compared our patterns of ILP phenotypes with published spatial and temporal patterns of ILP expression [31], [43]. Thus, these findings suggest that there is extensive regulatory and functional diversification in the ILP system that results in unique sets of functions for each ILP.

Previously, we and others have shown that certain ILPs could execute regulatory roles that are either similar or opposite to *daf-2* mutants [4], [5], [11], [27], [30], [39], [44]–[47]. This functional attribute extends to other ILPs. We found that 17 ILP mutants have phenotypes that largely resemble *daf-2* mutants, and may act as agonists in this pathway; while 4 ILP mutants (*ins-1*, *ins-3*, *ins-13* and *ins-20*) have phenotypes that are largely opposite to *daf-2*, and may act as antagonists. Lastly, 3 ILP mutants (*ins-12*, *ins-18* and *ins-23*) have similar or opposite phenotypes to *daf-2* in a process-dependent manner (Figure 1A). This last result suggests that the roles of some ILPs are context-dependent, which contributes to functional specificities.

Different phenotypes can be decoupled, implying considerable independence in their regulation. For example, the high-confidence hits for dauer entry differ from those for dauer exit (Figures 1A to 1E). The ILPs that regulate lifespan and pathogen resistance also do not overlap (Figures 1A and 1F versus 1H), even though increased immunity can contribute to the long life of *daf-2* mutants [33], [48]. We also observed decoupling of other processes, *e.g.*, lifespan versus thermotolerance (Figures 1A and 1F versus 1G), as well as dauer entry or exit versus pathogen resistance (Figures 1A and 1B to 1E versus 1H).

Many of the ILPs with detectable phenotypes are pleiotropic, indicating that they have diversified functions to coordinate multiple processes. The ILPs with high-confidence phenotypes in 2 or more processes (treating all 3 dauer entry screens as one process) constitute 52% (12/23), which become 81% (25/31) if low-confidence phenotypes are included. Pleiotropy allows one gene to coordinate multiple processes, but sacrifices specificity and independent regulation. To examine how these trade-offs and constraints are manifested in the ILP system, we quantified the extent to which the relationships between ILPs and their phenotypes are compartmentalized (modular) or intermeshed (non-modular) based on a measure of modularity from 1 to 0 [49]. A value of 1 indicates a perfectly modular system, where distinct sets of ILPs function without cross-regulation among modules. The modularity value of the ILP-to-phenotype map is 0.42, indicating that the ILP system is partly modular, constituting a compromise between independent regulation and coordination.

Together, these findings reveal the operational rules of a combinatorial code that links ILPs to phenotypes. Despite its prominence, redundancy is not a universal feature of the entire ILP system; instead, the ILPs are characterized by substantial functional specificity and diversity, providing mechanisms through which functional complexities arise in gene families.

### *C. elegans* ILPs Are Organized in a Gene Expression Network

**Figure 2:**
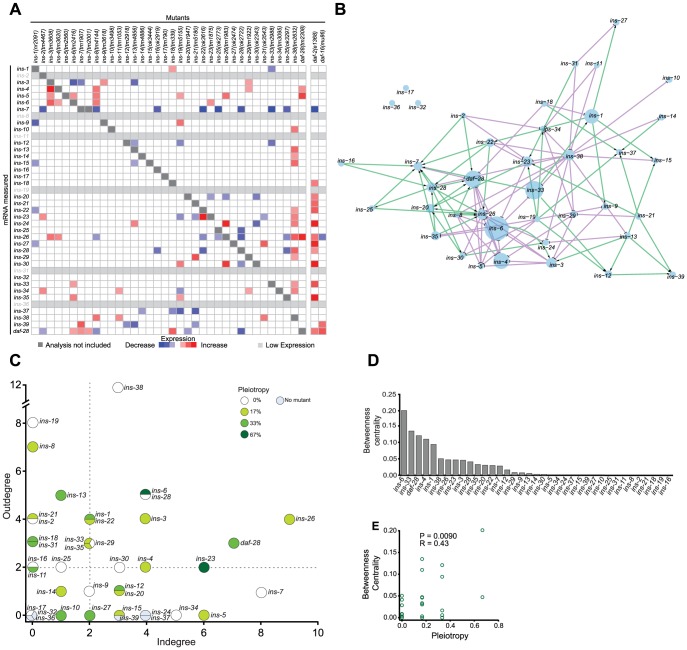
The ILP regulatory network. (**A**) Heat map summarizing the significance and direction of expression changes for all ILP mRNAs in 36 ILP mutants represented by negative log of the p-values. Significant differences (q<0.05) and transcripts with low expression were colored as indicated in the legend; more significant changes are more darkly shaded. Transcripts corresponding to deleted ILPs were excluded because these changes were not due to regulation (see Table S5 for mRNA changes). (**B**) Gene expression network based on a spring-embedded layout. Node sizes reflect the magnitude of betweenness centrality. Green and violet lines indicate excitatory and inhibitory connections, respectively. (**C**) A scatter plot of inputs versus outputs for each ILP. Pleiotropy signifies the percentage of phenotypes affected by each ILP; dauer entry phenotypes were aggregated for this purpose (see Materials and Methods and Figure 1). Dashed lines indicate the median for each axis. (**D**) Rank order of the ILPs for betweenness centrality. (**E**) Scatter plot showing linear correlation of betweenness centrality with pleiotropy.

ILP-to-ILP signaling regulates several physiological processes [4], [24], [30]. To investigate its global nature, we used quantitative real-time PCR (qPCR) to identify changes in the mRNA levels of all 40 ILPs in each of 35 ILP mutants (Figure 2A, S2, and Table S5). Surprisingly, we found that ILP-to-ILP signaling extends to many members of this family, demonstrating the presence of an ILP-to-ILP regulatory network (Figure 2A). Out of a possible 1190, we observed only 101 ILP interactions (Figure 2A), which suggests that the inter-ILP regulation is sparse. These regulatory relationships also appear specific and diverse: each ILP is wired to a unique combination of regulators and targets, and regulation could be either negative (52%) or positive (48%) in a target-specific manner. These relationships showed an intermediate modularity of 0.49, reflecting a mix of cross-regulation and compartmentalization in ILP gene expression. Thus, like the phenotypic screens, the qPCR data show that the diversification of *C. elegans* ILPs beyond functional redundancy also extends to their gene expression.

For comparison, we also analyzed the changes in expression of all 40 ILPs in mutants that impair the ILP signaling pathway, using *daf-2(e1368)* (a reduction-of-function allele), and *daf-16(mu86)* (a null allele) [18], [34]. Many ILPs were up-regulated in the *daf-2(e1368)* background, suggesting compensation. Many of the ILPs that were regulated by other ILPs were also affected in the *daf-2(e1368)* and *daf-16(mu86)* backgrounds, suggesting that these changes were mediated through the canonical ILP signaling pathway. In general, *daf-2(e1368)* and *daf-16(mu86)* tend to cause larger effects on gene expression, suggesting that they might be closer to the upper limit of the gene expression changes, as might be expected if the central pathway for ILP signalling is disrupted. Some ILPs that were regulated by other ILPs were not affected by *daf-2(e1368)* or *daf-16(mu86)*; this difference could be due to residual signaling activity retained in *daf-2(e1368)* [34] or the use of alternative pathways for inter-ILP regulation.

To understand inter-ILP communication, we built a network based on these qPCR results for graph theory analysis, treating each ILP as a node and each regulatory interaction as an edge (Figure 2B). In this network, the edges are directed (reflecting the regulation of one ILP by another) and signed (indicating positive or negative regulation) to represent the flow of information.

We discovered three major properties of this network. First, the ILP network had “small world” properties defined by two key parameters: the characteristic path length that measures the average minimal number of edges between all possible pairs of ILPs, and the clustering coefficient that measures the density of local interconnections [50], [51]. Compared with random networks with the same number of edges and nodes, the ILP network has a short path length, 3.17, and a high clustering coefficient, 0.13 (Figures S3A to S3C). Respectively, these properties might suggest that within these genetic circuits, signals can be communicated relatively efficiently from one ILP to another because they are separated by very few intervening ILPs, and that information is processed by local genetic circuits. These are consistent with the parallel processing we observed in the dauer entry sub-network, which is discussed below.

Second, the ILP expression network displayed hierarchical regulation. Plotting the number of regulators (in-degree) versus the number of targets (out-degree) of each ILP (Figure 2C) reveals a regulatory hierarchy where several ILPs had an exceptionally high number of regulators or targets. This organizational feature suggests different functional attributes for the ILPs. ILPs with few inputs and many outputs are putative upstream regulators; ILPs with similar numbers of inputs and outputs likely act in relays or processing circuits; and ILPs with many inputs and few outputs could serve as downstream integrators or effectors.

Third, important nodes for network communication tend to affect more processes. We calculated the betweenness centrality for each ILP, which measures its importance as a link between other ILP pairs in the network (Figure 2D) [52]. ILPs with higher betweenness centrality were more likely to be pleiotropic (Figures 2D to 2E), similar to protein-interaction networks where proteins with high betweenness centrality tend to be essential [52]. Thus, ILPs with high betweenness centrality may act as bottlenecks during information flux in a wider range of processes.

Our network analysis was robust to missing edges, such as those from subtle gene expression changes that did not rise to statistical significance. The top ranked ILPs for each network parameter were similar despite the addition or removal of 25% of random edges (Figures S3H to S3K), indicating that we have sampled the network sufficiently.

**Figure 3:**
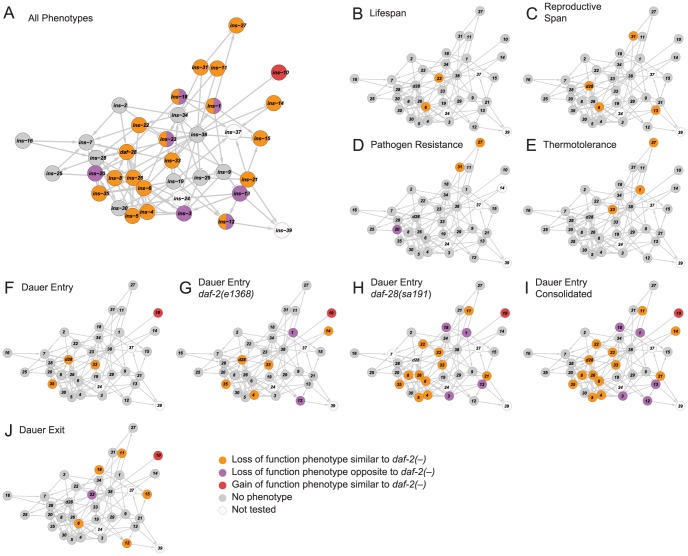
Functional maps of the ILP network. Phenotypes of the ILPs were mapped onto a spring-embedded layout of the ILP network; directions of phenotypes are indicated in the legend (bottom right). (**A**) shows the network where ILPs with phenotypes are highlighted based on their phenotypic direction compared to *daf-2* mutants. ILPs with both similar and opposite phenotypes to *daf-2* are indicated with a split circle. (**B–J**) shows the network highlighted for phenotypes as indicated.

To relate ILP function to network organization, we mapped the high-confidence ILPs identified in each screen onto the network, which provided three global observations. First, the ILPs with phenotypes were spread over the network (Figure 3A), suggesting that signaling across many parts of the network was important for its overall function. Second, the ILPs with more specific phenotypes from the non-sensitized screens were segregated into different locations (Figures 3B to 3F and 3J), consistent with the observations that gene expression defects in these ILP mutants do not propagate over the entire network (Figure 2A). The separation of critical nodes in the network could limit the number of physiological defects when one ILP is perturbed. Third, our sensitized screens for dauer entry revealed another functional level of non-critical ILPs distributed over much of the network (Figures 3F to 3I). This suggests distributed processing, which could reduce the severity of a phenotype by providing alternate routes of communication. Together, these mechanisms contribute to functional specificity, which is an aspect of the ILP combinatorial code.

### Diverse Genetic Interaction Profiles among ILPs

**Figure 4:**
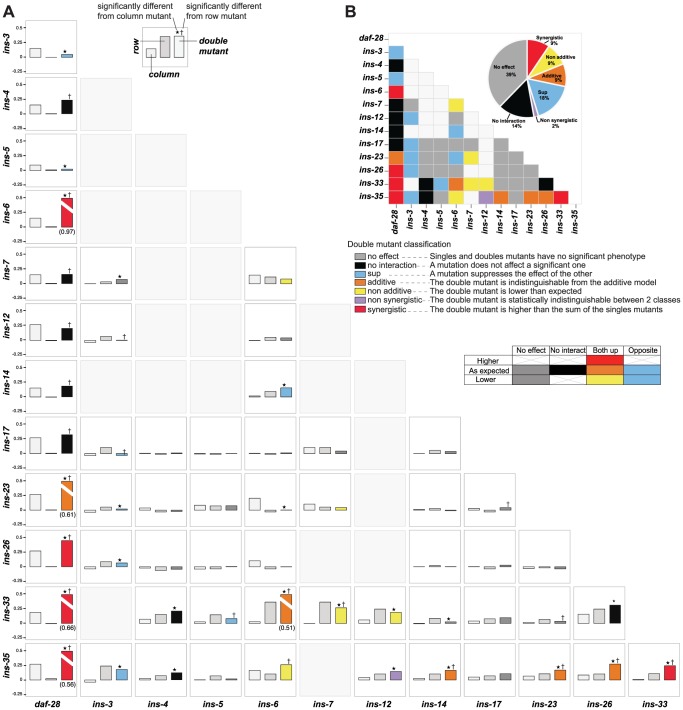
Genetic interactions between ILPs. (**A**) Double mutant analysis for dauer entry. Bars represent the phenotypes of single and double mutants (legend at bottom right); the class of genetic interaction is colored in the bar for the double mutant. (*) and (†) indicate significant difference with the column and row mutant, respectively (p<0.05, hypergeometric test). (**B**) Heat map and a pie chart summarizing the different genetic interactions based on dauer entry phenotypes of single and double mutants. Percentages in the pie chart do not add up to 100% due to rounding errors.

To address how ILPs combinatorially regulate a specific process, we analyzed genetic interactions among deletion mutations of ILPs involved in dauer entry. We tested 56 double mutant combinations by selecting a diverse subset of 13 ILPs identified from each of the three dauer entry screens, encompassing ILPs showing high and low penetrance (Figures 1B to 1D). To classify genetic interactions, we first determined how the fraction of dauer entry in the double mutant differed from the expected fraction in an additive model based on the single mutant phenotypes (Materials and Methods). We then subdivided the interactions based on whether the corresponding single mutants had the same or opposite phenotypes (Figure 4, Table S6). This analysis revealed a level of diversity in gene interactions not predicted by simple redundancy.

Diverse genetic interactions (defined in Figure 4) were observed in 47% (26/56) of the double mutants, of which 38% (10/26) were additive or synergistic. This result indicates that while the choice between dauer arrest and reproductive growth is binary, the likelihood of a given choice is specified by a graded combination of ILP activities. Strikingly, 9 of these 10 additive or synergistic interactions were seen in double mutants with null mutations in either *ins-35* or *daf-28*, suggesting that these ILPs are important genetic hubs in dauer entry, consistent with their strong dauer entry phenotypes. The remaining 53% (30/56) of the double mutants showed no effect or no interaction (Figure 4, Table S6), indicating that ILPs are not promiscuous in their interactions during dauer entry, even with other ILPs involved in the same process. These results reveal how signals from pairs of ILPs are integrated to regulate dauer entry. Our findings also demonstrate functional differences among ILPs that regulate dauer entry, and indicate that the effect of an ILP depends on genetic background.

### Information Processing in the Dauer Entry Sub-network

**Figure 5:**
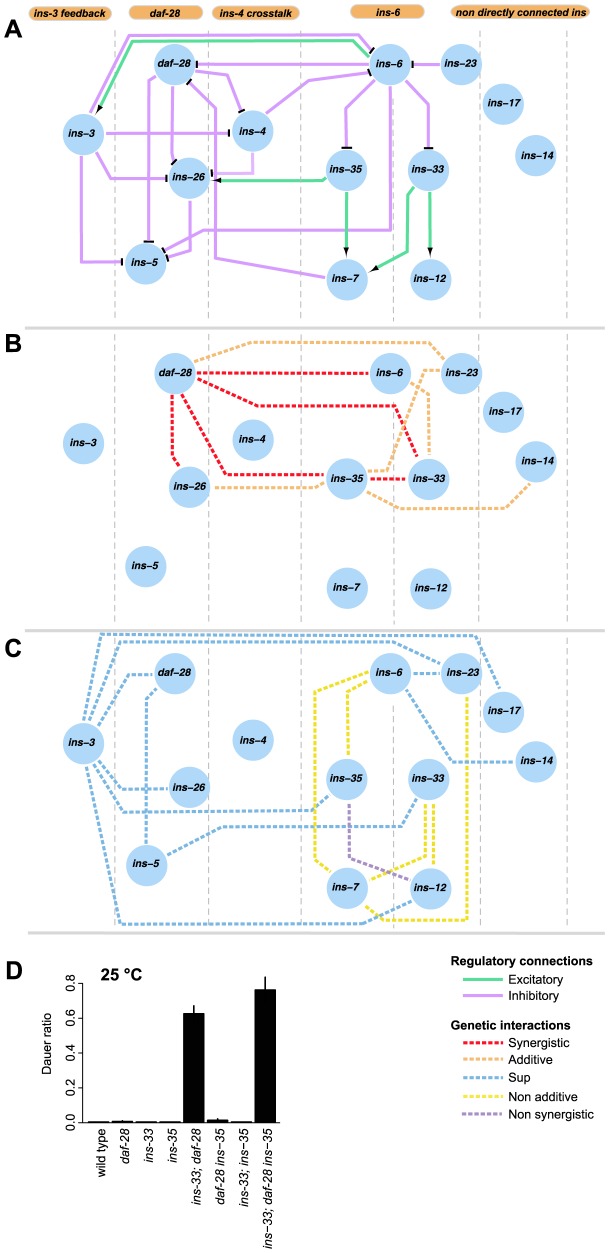
Information flow in the dauer entry sub-network. (**A**) shows the putative organizational structure of the dauer entry sub-network comprised of dauer entry-regulating ILPs compared to their genetic interactions (**B, C**). Genetic interactions classified as additive or synergistic (**B**); or non-additive, suppression or non-synergistic (**C**). (**D**) Shows the ratio of dauer formation of the triple mutant *ins-33; daf-28 ins-35* compared to double or single mutant strains. *ins-33; daf-28* and *ins-33; daf-28 ins-35* mutants are significantly more likely to enter dauer than all other strains (p<0.001, Hypergeometric test).

Information processing is strongly influenced by the signaling motifs within the network and the overall network architecture [53]. While regulatory interactions serve as a roadmap for information flow among ILPs, genetic interactions between ILPs reflect how their activities are integrated to generate a physiological outcome. To assess information flow and processing, we combined regulatory and functional data for the ILPs whose genetic interactions were extensively defined for the dauer entry phenotype (Figure 5).

The connectivity and synergistic or additive genetic interactions indicate parallel signaling in the dauer entry sub-network (Figures 5A to 5B). The major signals that inhibit dauer entry come from three main branches (*daf-28*, *ins-6/ins-33* and *ins-6/ins-35*), because mutants in these branches have the strongest phenotypes (Figure 1C). To generate graded probabilities of dauer entry, signals from these three branches are integrated in an additive or synergistic manner based on their genetic interactions (Figures 4 and 5B). This network organization was supported by the phenotypes observed when we disrupted the *daf-28*, *ins-6/ins-33* and *ins-6/ins-35* branches using combinations of null mutations. In the *ins-33* and *daf-28* double deletion mutant, we observed a strong synergistic response with a high proportion of dauers even at 25°C (Figure 5D, Table S7). Strikingly, in the *ins-33; daf-28; ins-35* triple deletion mutant, up to 80% dauers were observed at 25°C (Figure 5D, Table S7), which is nearly comparable to *daf-2* mutants. These results reinforce the idea that the *daf-28*, *ins-6/ins-33* and *ins-6/ins-35* branches are major pathways for regulating dauer entry.

The different connectivities within each branch of the ILP network suggest that they use different information processing strategies (Figure 5). In the *daf-28* branch, *daf-28* inhibits *ins-26*, which likely serves as a compensatory regulation based on their synergistic interaction (Figures 4 and 5B). The effect of this compensation is likely to be regulation of *ins-5* as both *daf-28* and *ins-26* inhibit *ins-5*. In contrast, the *ins-6* branches have a bifurcated topology where *ins-33* and *ins-35* process inputs from *ins-6*. A non-additive interaction was observed between *ins-6* and *ins-35*, as well as between *ins-6* and *ins-7*, which is downstream of *ins-35* (Figures 4 and 5C); while an additive interaction between *ins-6* and *ins-33* indicates compensation (Figures 4 and 5B; see below). At a downstream level, non-additive or non-synergistic interactions occur within the *ins-33* or *ins-35* branches, but not the *daf-28* branch. Crosstalk occurs between the *daf-28* and *ins-6* branches (Figure 5A), which may coordinate their signaling activities.

*ins-3* is likely to act as a negative modulator providing feedback to the dauer entry sub-network at multiple levels; such circuits are associated with noise reduction and homeostasis. Unlike most ILPs, the *ins-3* mutation decreased dauer entry in several backgrounds (Figures 1D and 4). *ins-3* expression was activated by *ins-6*; while *ins-3* in turn inhibited *ins-6* expression, as well as other ILPs in the *daf-28* branch (Figures 2A and 5A).

While both *ins-14* and *ins-17* show high and low-confidence dauer entry phenotypes, respectively, they are likely to act separately as modulators in the main dauer entry sub-network (Figure 5) for two reasons. First, they are not directly connected to the *ins-6* and *daf-28* branches of the expression network (Figures 5A to 5C). Second, they have weaker interactions with the genes in the *daf-28* and *ins-6* branches (Figure 4A). One exception is an additive interaction between *ins-35* and *ins-14* (Figures 4 and 5B), which might represent cross-talk at the downstream level.

### A Regulatory Mechanism for Phenotypic Specificity

ILPs could also exert either strong or weak effects (Figure 1). For example, although *ins-6* and *daf-28* both regulate dauer entry and exit, *ins-6* null mutations had a stronger effect on dauer exit, whereas *daf-28* null mutations had a stronger effect on dauer entry [5]. Our results reveal that this feature is common in the whole ILP system (Figure 1). These specificities are not due to some ILPs being generally strong signals, while others are generally weak, because the relative effects of the ILPs can be reversed depending on the phenotypes.

Our integrated analysis provided a mechanistic explanation for the phenotypic specificity of *daf-28* and *ins-6* (Figure 1C) during dauer entry. Loss of *daf-28* is compensated by *ins-26*, because *ins-26* was up-regulated in *daf-28* mutants (Figures 2A and 5A) and because *ins-26; daf-28* double mutants have a more severe phenotype than either single mutant (Figures 4 and 5B). However, *ins-26* is a weak compensator, as indicated by its weak phenotype (Figure 1C). Additionally, *daf-28* mutants up-regulate *ins-5* (Figures 2A and 5A), an ILP that can promote dauer entry (Figures 1 and 4). As opposed to compensation, increased *ins-5* expression contributes to the mutant phenotype of *daf-28*, because removing *ins-5* suppressed the *daf-28* mutation (Figures 4 and 5C). These two targets of *daf-28* therefore contribute to its strong dauer entry phenotype.

In contrast, *ins-6* is compensated by *daf-28* and *ins-33*, because both *daf-28* and *ins-33* were up-regulated in *ins-6* mutants (Figures 2A and 5A), and both *ins-6; daf-28* and *ins-33; ins-6* double mutants had a more severe phenotype than the respective single mutants (Figures 4 and 5B). Both *daf-28* and *ins-33* were strong compensators, as indicated by their strong phenotypes (Figure 1C). Thus, the weak *ins-6* phenotype could be explained by compensation from two strong regulators.

Together, these results show that connectivity within the ILP network serves as an important determinant of functional differences among ILPs.

## Discussion

Most animals, including humans, encode multiple ILPs in their genomes, which regulate multiple processes [4], [5], [14], [15], [23], [24], [26], [27], [54]–[58]. However, the biological function of large ILP ensembles remains an open question. Our systematic analysis of *C. elegans* ILPs revealed that they are organized into an ILP-to-ILP network that provides several regulatory mechanisms for graded signaling, functional diversity, robustness to gene perturbation and information flow. In turn, these functional properties of the ILP network generate aspects of a combinatorial code that links ILPs to developmental and physiological outputs. Thus, our findings challenge the notion that broad redundancy is the central feature of the *C. elegans* ILP family.

Large gene families are often proposed to employ a combination of redundancy and diversity to regulate biological processes [59]. Here, we reveal the specific implementation of an ILP combinatorial code that coordinates aspects of development and physiology (Figure 1A). Different ILPs generally affect different combinations of processes, which support the idea that redundancy is not evolutionarily stable unless the genes have additional functions [59], [60]. The high-confidence phenotypes indicate that many single ILPs can significantly contribute to different phenotypic outputs. This combinatorial coding of phenotypes therefore argue against simple redundant mapping between ILPs and their outputs, but show that the complexity of these gene-phenotype relationships is generated at least in part by inter-ILP communication.

The intermediate modularity of the ILP phenotypes raises the possibility that multiple ILP signaling centers exist in the animal, which could provide differential contributions to different processes. In addition to the regulatory connectivity that underlies phenotypic specificity, spatial specificity in ILP signaling could also be a complementary mechanism in achieving the specific patterns of ILP phenotypes. This model will need to be tested in the future by tissue- or cell-specific rescue of the ILPs, coupled with the elucidation of their downstream target tissues where the DAF-2 ILP receptor acts.

Undirected networks have been recently used to group the *C. elegans* ILPs based on similarities in their expression patterns [31]. Here we show that the *C. elegans* ILPs are organized at the level of ILP-to-ILP regulation in a directed regulatory network, where signals in different branches are processed differently and modulated by cross-talk. This is exemplified in the different connectivities between the *ins-6* and *daf-28* branches of the dauer entry subnetwork, whose distinct signals are ultimately integrated to set the probability of dauer entry. This network organization thus contributes to the graded nature of the ILP combinatorial code. This property generates different probabilities of dauer entry that result in different fractions of developmentally arrested dauers versus reproductive adults within a population. Dauers can survive environmental insults that kill reproductive adults and can thus serve as a hedge at the cost of delayed reproduction. Therefore, the advantage of this graded response provided by the parallel circuit organization is the ability to optimize the trade-off between fast reproduction versus survival in response to variable environments.

These findings further underscore how circuit organization in a network contributes to the phenotypic outputs of a multi-gene family. Compensation and distributed, parallel processing in the ILP network provide robustness against gene or network perturbation. Robustness in preventing dauer entry allows for rapid reproduction, ensuring that animals develop as dauers only in extreme conditions, such as when the environment impinges on more than one ILP.

In addition, the connectivity of the ILP network show that specific compensatory circuits are organized to generate strong and weak regulators, an important component of the combinatorial code. Extensive genome-wide studies in yeast indicate that complete or partial functional redundancy can occur among duplicated gene pairs [61], [62] where the loss of one gene can be compensated by responsive circuits that increase the expression of a second homologous gene [63]. Although compensatory circuits are often hypothesized as a feature of gene families that lead to redundancy, we show that its actual implementation can lead to more complex outcomes than previously proposed. Instead of global redundancy, the gradation provided by the ILP network is consistent with the idea that partial redundancy, as well as overlapping and distinct functions, could serve to encode diverse inputs [59], [60].

ILP-to-ILP signaling in diverse animals uses similar signaling motifs, such as feedback, compensatory inhibition and feedforward circuitry [4], [24], [28]–[30], [64], [65], which may provide similar biological functions despite component differences [53]. Our findings suggest how simple circuits can be organized to generate complex network functions; like signaling motifs, these principles may also apply to networks in general. Because our results indicate the importance of specificity versus redundancy in multi-gene families is a consequence of network organization, we propose that large-scale connectivity-based approaches have general utility in dissecting the regulatory mechanisms employed by different families of intercellular signals in different animals.

In summary, we have delineated the *C. elegans* ILP-to-ILP regulatory network based on functional criteria, which provides a distinct approach to existing ILP networks based on expression similarities [31]. This ILP-to-ILP regulatory network, coupled with our systematic genetic analyses, serves as a mechanistic framework for understanding information processing by ILPs. Our findings suggest that the multiple ILPs provide the ability to organize circuits into a network with diverse points of regulation, which in turn produces an intricate combinatorial code to orchestrate development and physiology. Together, this represents a new avenue to understand how hormonal systems compute the development and physiology of the organism.

## Materials and Methods

### *C. elegans* Strains and Culture

*C. elegans* were cultivated at 20°C under standard conditions except where otherwise stated. The strains used are listed in Table S1. All ILP deletions were independently confirmed using PCR from genomic DNA with primers different from those used by the *C. elegans* Knockout Consortium to isolate the mutation. *ins-10(tm3498)* had increased expression of the coding region from our qPCR experiments (below). PCR using genomic DNA from 6× outcrossed *ins-10(tm3498)* mutants with primers that annealed to the start and end of the *ins-10* coding sequence amplified a genomic fragment that contained the full *ins-10* coding sequence which was verified by sequencing (data not shown). Because *ins-10(tm3498)* also contained a deletion in the endogenous *ins-10* locus, which we verified independently from the *C. elegans* Knockout Consortium, these results indicate that *ins-10(tm3498)* involves at least a deletion and duplication of the *ins-10* coding region that led to *ins-10* overexpression.

All mutant strains used in this study were obtained from the Knockout Consortium [66]. Double and triple mutants were generated by standard genetic methods. See Table S1 for strain list. Deletions were regularly verified using PCR.

### Phenotypic Analysis

All the phenotypic assays were conducted on fresh NGM plates seeded with fresh OP50 unless specified otherwise, using animals that were well fed for at least 2 generations. The lifespan and dauer assays were replicated in different labs. The identity of each strain was blinded for most assays.

#### Lifespan

Life spans were performed at 20°C as previously described [67]. No RNAi or drug treatments were used during the assay.

#### Dauer entry and exit

Dauer entry and exit assays were also performed with minor modifications to previous descriptions [5]. For dauer entry assays, worms were allowed to lay eggs at 20°C for 4 hours; 50 eggs were distributed per plate, incubated at the temperature specific for each experiment and scored 48 hours later. For dauer exit assays, dauers induced by the *daf-2(e1368)* mutation at 25°C were distributed at 25–50 per plate, kept at 25°C and scored at 12 hour intervals for 10 days.

#### Pathogen resistance

Pathogen resistance was tested using the slow killing kinetics assay that is similar to one described previously [42]. Briefly, ∼5 µl overnight Luria Broth culture of the *Pseudomonas aeruginosa* clinical isolate strain PA14 was spread on an NGM plate to make a lawn of ∼5 cm^2^ and the inoculated plate was incubated at 37°C for 24 hours before use. Subsequently, 20 L4-stage hermaphrodites were transferred onto each PA14-plate. The plates were kept at 25°C and scored for live worms every 8–9 hours. Live worms were transferred to a freshly prepared PA14-plate on every other day to prevent progeny contamination. We did not use any pharmacological reagents or RNAi treatment during the slow killing assays.

#### Reproductive spans

Reproductive spans were performed as previously described [68], except that they were performed at 15°C, and progeny checks were done three days after removing the mother.

#### Thermotolerance

Automated survival assays were conducted using the *C. elegans* lifespan machine [41]. Wild-type and mutant eggs were collected via hypochlorite treatment of adults grown at 20°C, and placed at 20°C on NGM-agar plates seeded with OP50. Late-L4 larvae (approximately 48 hours post bleach) were transferred onto fresh plates containing 13.3 µg/mL 5-fluoro-2′-deoxyuridine (FUDR, Sigma). Day 3 adults (approximately 72 hours post late L4) were transferred onto fresh plates (NGM-agar without CaCl_2_, to ensure clarity of agar) at a density of 35 worms per plate. These plates were placed onto modified flatbed scanners calibrated to operate at 34.5°C. Death times were automatically detected by the lifespan machine's image-analysis pipeline [41], and survival distributions were validated through visual inspection of representative subsets of collected image data. Rescue experiments were conducted manually, where worms were pre-treated as above and the Day 3 adults were transferred onto fresh NGM-agar plates at a density of 10 animals per plate. These plates were then shifted to 34.5°C and assayed for survival every hour, until all animals have died.

#### Dauer entry for double mutants

Dauer entry phenotypes were assayed at 27°C, except for experiments involving *daf-28* mutants. *daf-28(tm2308)* mutants have near saturating rates (∼80–90%) of dauer entry at 27°C, precluding accurate estimates of potential additional effects in double mutants; for a greater dynamic range, strains with *daf-28(tm2308)* were tested at 26.5°C.

We note that some mutants showed variable effects on certain phenotypes (Figure S1). In particular, *ins-35* and *ins-38* showed significant changes in dauer exit rates, but were highly variable either between trials or between labs. Perhaps these mutants are particularly sensitive to environmental variables that cannot be easily controlled, or they increase phenotypic variability rather than simply regulating a specific phenotype.

#### Rescue experiments

With the exception of *ins-12*, where we used a stable integrant, we tested for rescue of the respective phenotypes by comparing animals harboring an extrachromosomal array containing genomic regions of the gene of interest with siblings lacking the array. Lines bearing or lacking the array were selected by the presence or absence of the *ofm-1::gfp* marker [5]. For some ILPs, not all transgenic lines showed rescue in all trials; this might be due to variability in gene expression, gene silencing, gene dosage or mosaicism from extrachromosomal arrays. A control strain containing only the *ofm-1::gfp* co-injection marker did not show any effects on lifespan, thermotolerance or dauer entry (data not shown).

### Molecular Biology and Generation of Transgenic Lines

We generated plasmids to rescue the phenotypes of the ILP mutants. These plasmids contain the entire coding region of the gene of interest and the 5′ and 3′ intergenic regions up to the next open reading frame. Genomic regions for *ins-3*, *ins-4*, *ins-5*, *ins-14*, *ins-15*, *ins-21*, *ins-23*, *ins-26*, and *ins-27* were subcloned using a recombineering method [69] from the corresponding fosmids into the pQL60, a vector derived from the original pPUB in which the *unc-119* marker was removed. Genomic regions for *ins-31*, *ins-33* and *ins-35* were amplified by PCR and subcloned into pCR-Blunt TOPO (Invitrogen). The transgenic lines bearing extrachromosomal arrays were generated by microinjection of the rescue construct at different concentrations (see Table S1) as well as *ofm-1::gfp* as a coinjection marker (25 ng/µl) and pBluescript as a carrier DNA up to a final concentration of 100 ng/µl of DNA.

For *ins-12*, a mini-gene was synthesized, subcloned into the MosSCI plasmid pCFJ352 [70] with the corresponding the 5′ and 3′ intergenic regions up to the next open reading frame and integrated into the QL35 strain using MosSCI [71].

### Statistical Analysis

#### Phenotypic analysis

The log rank test based on right censoring was used to identify significant differences in survival functions for lifespan, dauer exit, thermotolerance, and reproductive span between control and experimental strains. The log rank test based on intervals censoring was used to determine significant changes in pathogen resistance. The hypergeometric test was used to determine significant changes in the various dauer entry assays. Depending on the statistical test, we considered three possible criteria for classifying the screening results: (1) considering all trials for a given mutant, the p-value determined by summing the chi-square statistics (for log-rank tests) or a linear model (for hypergeometric tests) was less than 0.01 for the corresponding degree of freedom ( = number of trials); (2) if the mutant was tested in two labs, it had to be significant (p<0.05) in the majority of trials in both labs; and (3) if the mutant was tested in one lab, it had to be significant (p<0.05) in the majority of trials and be significant in at least in 2 trials. A high confidence hit was assigned if all three criteria were met; otherwise, a low confidence hit was assigned if criteria (1) was met. A variable hit was assigned when significant trials were observed in both directions (Figure S1). Statistical tests and classification of high and low confidence hits were implemented in R. Heat maps were visualized using Javatree. Correlation analysis between expression and phenotypic patterns were performed in R.

#### qPCR analysis

Data were normalized against the geometric mean of the two most stable reference genes identified by geNorm, *pmp-3* and *Y45F10D.4*. The statistical significance of differences in ILP mRNA levels between wild type and each mutant (3–4 replicates each) were determined with p-values obtained from a linear model and corrected for multiple hypothesis testing using a q-value (false discovery rate) threshold of 0.05 for this dataset [72]. Six ILP mRNAs were expressed at low or undetectable levels and not analyzed further. We used custom scripts in Igor Pro 6 and the qvalue package in R (http://www.r-project.org/). We tested 2 alleles of *ins-7*; although we report all interactions in Figure 2, only the interaction common to both alleles were considered in our subsequent analysis.

#### Double mutant analysis

To estimate the probability of dauer entry of a double mutant (P_xy_), using the measured probability of the dauer entry in each single mutant (P_x_ and P_y_) if the two mutations acted independently, we used the equation: 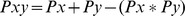. Using P_xy_, we calculated the number of dauers and non-dauers expected, if we had scored the same number of animals as we did for the double mutant. We next determined if the actual number of dauers and non-dauers significantly deviated from expectation (hypergeometric test, p<0.05) on a trial by trial basis. The majority outcome in multiple trials was used to classify each double mutant into 3 groups: significantly higher than expected, within expectation if there was no interaction, or significantly lower than expected. The interactions that were higher than expected were classified as synergistic. The remaining 2 groups were further subdivided based on the differences between wild type versus single mutants, wild type versus the double mutant, and each single mutant versus the double mutant, using essentially the same statistical analysis performed for the phenotypic analysis of the single ILP mutants; differences were deemed significant when at least half of the trials showed p<0.05 (hypergeometric test). After ruling out cases where there were no genetic interactions or no phenotypes, the interactions that were not higher than expected were subdivided into three classes. The first class consisted of cases where the two single mutants have opposing effects on dauer entry across the dataset. *ins-3* and *ins-5* tended to reduce dauer entry frequencies and this class largely consisted of double mutants containing either of these mutations. Examining this class revealed whether *ins-3* or *ins-5* could suppress the increased dauer phenotypes found in the other mutant. The second class consisted of cases where both single mutants led to increased dauer entry rates. Examining this class indicated whether the increased dauer rates were additive or not. The third class consisted of the *ins-12; ins-35* double, which was significantly different from *ins-12* and not *ins-35*, but was not significantly different from expected if the interactions were additive. Because these results made it difficult to unambiguously classify the interaction as additive or non-additive, we classified it conservatively as non-synergistic.

### qPCR and Network Analysis

#### Sample preparation

Synchronized L4s (between 160–200 individuals) were picked, aged to adulthood and transferred to OP50-seeded 14 cm plates, where they were allowed to lay eggs at 20°C for 4 hours and removed from the plate afterwards. Plates were incubated at 20°C for 52 hours, until the animals reached the L4 stage. Worms were then washed away with ice-cold M9, collected into Falcon tubes and centrifuged for 2 minutes at 400 RCF and 4°C. Worms were then washed 3 times with ice-cold M9 to remove all traces of bacteria. Worms were then transferred to RNAse-free Eppendorf tubes and resuspended in 50 µl of RNAlater RNA stabilization solution (Qiagen). The worms were stored at −80°C till further use.

#### RNA extraction

Before proceeding with the RNA extraction, the RNAlater was removed by centrifugation at maximum speed for 10 min at 4°C. RTL (250 µl, RNeasy Mini Kit, Qiagen) and 250 mg of glass beads were added to the samples. The worms were lysed using a TissueLyzer (Qiagen) at 20 Hz for 2 min and the procedure was repeated for 1 min. Samples were kept in ice whenever possible. After centrifugation of the lysed samples, the supernatants were transferred to a new RNAse-free Eppendorf tube and 1 volume of 70% ethanol was added. The procedure was then continued as described by the manufacturer (RNeasy Mini HandBook, Qiagen). The subsequent experimental steps for the qPCR analysis were performed by qStandard (UK).

#### RNA and assay quality controls

RNA integrity was assessed using an Agilent Bioanalyzer and samples with RIN<1.8 were rejected. RNA concentration and purity were determined using a NanoDrop spectrophotometer. Samples were used only if peak absorbance occurred at 260 nm, A260/280>2.0, A260/230>1.0, and RNA concentration was at least 200 ng/µl. Two micrograms of RNA were reverse transcribed using a Quantitect reverse transcription kit (QIAGEN UK) in a 20 µl reaction volume and included a gDNA wipeout step, with 10% of reactions performed in duplicate. Complementary cDNAs were diluted 10-fold with yeast tRNA (5 ug/ml); 2 µl of this was used for each qPCR reaction. Assays were designed by qStandard and tested for specificity by running products amplified from cDNA on a 2% agarose gel. Primers (Table S2) were free from known SNP sites and both primers and amplicon sequences were analysed using M-Fold software to avoid potential secondary structure. A standard of known copy number, calculated using the specific extinction coefficient for each amplicon, was generated for each assay from purified PCR products; only assays that exhibited sensitivity to 10 copies/reaction, linearity over 7 log and efficiency ≥95% were used.

#### qPCR

Two microlitres of cDNA were amplified in a 10 µl reaction using Quantifast SYBR green master mix (Qiagen) with each primer at a final concentration of 500 nmol/l. Control reverse transcription-negative reactions were run with primers for an intron-flanking assay, capable of detecting gDNA (*ins-2*) and the completed reactions were run on a 2% agarose gel. No significant gDNA contamination was observed, indicating effective gDNA removal at reverse transcription. For all assays, template controls and DNA standards (101–107 copies/reaction) were included in each run. Amplification parameters were 95°C for 5 min followed by 40 cycles of 95°C for 10 sec, 57°C for 20 sec and 72°C for 10 sec, using a Rotor-Gene 6000 (Corbertt). Melt curves were checked for product specificity (single peak) and the presence of primer dimers. Primers were designed based on sequences obtained from www.wormbase.org and the primers sequences can be found in Table S4. Copy numbers per reaction were derived from the standard curves using the Rotor-Gene software.

#### Network construction

For network construction we considered only experiments where mRNA levels were robustly detected and interactions with q-value smaller than 0.05. The sign was determined by whether the target mRNA was upregulated or downregulated by the normal function of the ILP. We thus built a directed network based on significant changes in each gene's expression in each mutant. This regulatory network represents the gene expression interactions among 37 ILPs, as 3 ILPs (*ins-17*, *32* and *36*) showed no connections.

#### Small worldness

To calculate the small world coefficient of the network, we used the small world measure described in [73]. We calculated network small worldness using both clustering coefficient and transitivity, and obtained very similar small-world values (Figure S3A–C). To generate the random networks for the bootstrapping calculation and for calculating betweenness centrality, clustering coefficient and closeness centrality, we used pre-existing algorithms implemented in NetworkX [74].

#### Robustness analysis

To identify genes in key positions in the network, we ranked all the nodes in the network according to the following network measures: degree, betweenness centrality, closeness centrality and clustering coefficient. We then verified the robustness of this ranking by adding noise to the network in the form of addition or removal of extra edges, and re-calculating the ranking of every node after each iteration (Figure S3H–K) [75]. All network measures were calculated using NetworkX [74].

#### Visualization and phenotypic relationships

Networks were visualized using Cytoscape, including layouts, and displays of node and edge properties. Linear correlations were calculated in Igor Pro 6. Besides betweenness centrality (Figure 2D), we did not detect significant correlations between phenotype and network parameters, such as closeness centrality (which measures how many edges separate an ILP from all the other ILPs in the network), or between phenotype and clustering coefficient (which measures the amount of local interconnections), suggesting that these parameters are less crucial to ILP function (Figures S3D to S3G).

### Modularity Analysis

Modularity of the ILP-to-phenotype and the mRNA-to-ILP matrices were estimated by rearranging the rows and columns of the matrix to find highly interconnected groups and then assessing matrix-wide the ratio of the number of inside to outside group connections. We used the adaptive BRIM (Bipartite Recursively Induced Modules) algorithm [49], [76], which is a heuristic method, implemented in MATLAB [49], [76] to maximize a bipartite modularity value Q. This Q value is dependent on modularity of the matrix; by definition, a perfectly modular matrix is comprised of clusters of completely isolated groups (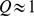), and modularity declines as the number of cross-group connections increases (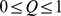). Because the modularity calculation is based on a stochastic algorithm that produced different matrix arrangement each time the algorithm is run, we performed the calculation 30 times and took the average of the modularity. The average modularity value of ILP-to-phenotype matrix is 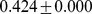 (highly reproducible) and that of mRNA-to-ILP is 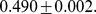 To evaluate the statistical significance of the modularity, we utilized two null models. The first model is a Bernoulli random null model in which the null matrix has the same total number of interactions as the original matrix, albeit randomly positioned. The second is a probabilistic degree null model in which each interaction in null model is assigned a probability. The ILP-to-phenotype and mRNA-to-ILP matrices are significantly different against the Bernoulli random null model (p<0.001 in both cases); however, when compared against the probabilistic degree null model, which is a stronger statistical test, the p-values of both matrices are greater than 0.05. These results suggest that both matrices are weakly modular.

## Supporting Information

Figure S1 Trial to trial variation in ILP phenotypes. The bar graphs indicate the mean magnitude of each phenotype normalized to same trial controls. The symbols within the bars represent the magnitude for each trial. Phenotypes tested were indicated in the Y-axis: (**A**) lifespan, (**B**) dauer entry, (**C**) dauer entry in the *daf-2(e1368)* background, (**D**) dauer entry in the *daf-28(sa191)* background, (**E**) dauer exit, (**F**) thermotolerance, (**G**) pathogen resistance, and (**H**) reproductive span. Bars and symbols were colored as indicated in the legend (bottom right).
(PDF)

Figure S2 qPCR expression graphs. The graphs represent the fold difference expressions compared to the wild type. Statistical significant changes are highlighted in red.
(PDF)

Figure S3 Network robustness and small world properties. Distributions obtained by bootstrapping analysis for (**A**) shortest path length, (**B**) clustering coefficient, and (**C**) small worldness based on the shortest path length and clustering coefficient. The values for the ILP network are indicated by a red vertical line in (**A–C**). Percentile of the ILP network compared to the random distribution is indicated for small worldness. (**D–E**) Rank order of the ILPs for clustering coefficient (**D**) and closeness (**E**). (**F–G**) Scatterplot of clustering coefficient (**F**) and closeness (**G**) versus pleiotropy as defined in Figure 2 along with P and R values for linear correlation. (**H–K**) The mean and standard deviation of the ranking after robustness analysis of each gene according to (**H**) degree, (**I**) betweenness centrality, (**J**) clustering coefficient, and (**K**) closeness centrality.
(PDF)

Table S1 Strain list.
(XLS)

Table S2 Summary of data from all screens.
(XLSX)

Table S3 Rescue experiments. For each experiment, the strain used for each rescue experiment is indicated in parenthesis after the genotype tested. **Effect** refers to the *p*-value significance difference between the mutant versus the control strains. **Rescue Effect** refers to the *p*-value significant difference between the mutant strain with and without a rescuing transgene. **Rescued Effect** indicates the *p*-value significance difference between the mutant strain with a rescuing transgene and the control strain. (*) For the *ins-14* rescue experiments, data from two trials (DA R3 and DA R4) were pooled together to obtain sufficient power for statistical analysis.
(XLSX)

Table S4 qPCR primer list.
(XLSX)

Table S5 qPCR data. Statistical analysis of the qPCR showing the T-statistics from a linear model, the q-value, the p-value and the means and SEMs normalized to wild type.
(XLSX)

Table S6 Summary of double mutant interactions in dauer entry. Positive and negative p values represent cases where the mutant dauer entry proportion is larger or smaller than the control, respectively. Significant p values (at 0.05 level) are coloured in pink or blue for higher or lower fractions of dauer, respectively.
(XLSX)

Table S7 Dauer analysis of the *daf-28; ins-33; ins-35* triple mutant strain. Statistical analysis of the differences in rates of dauer entry among strains with all possible combination of the 3 genes.
(XLS)
