# Supplementary figures and images for: An Insulin-to-Insulin Regulatory Network Orchestrates Phenotypic Specificity in Development and Physiology
Source: PLoS Genet. 2014 Mar 27;10(3):e1004225. doi: 10.1371/journal.pgen.1004225 (PMC3967928; doi:10.1371/journal.pgen.1004225)

Supplemental Figure 1

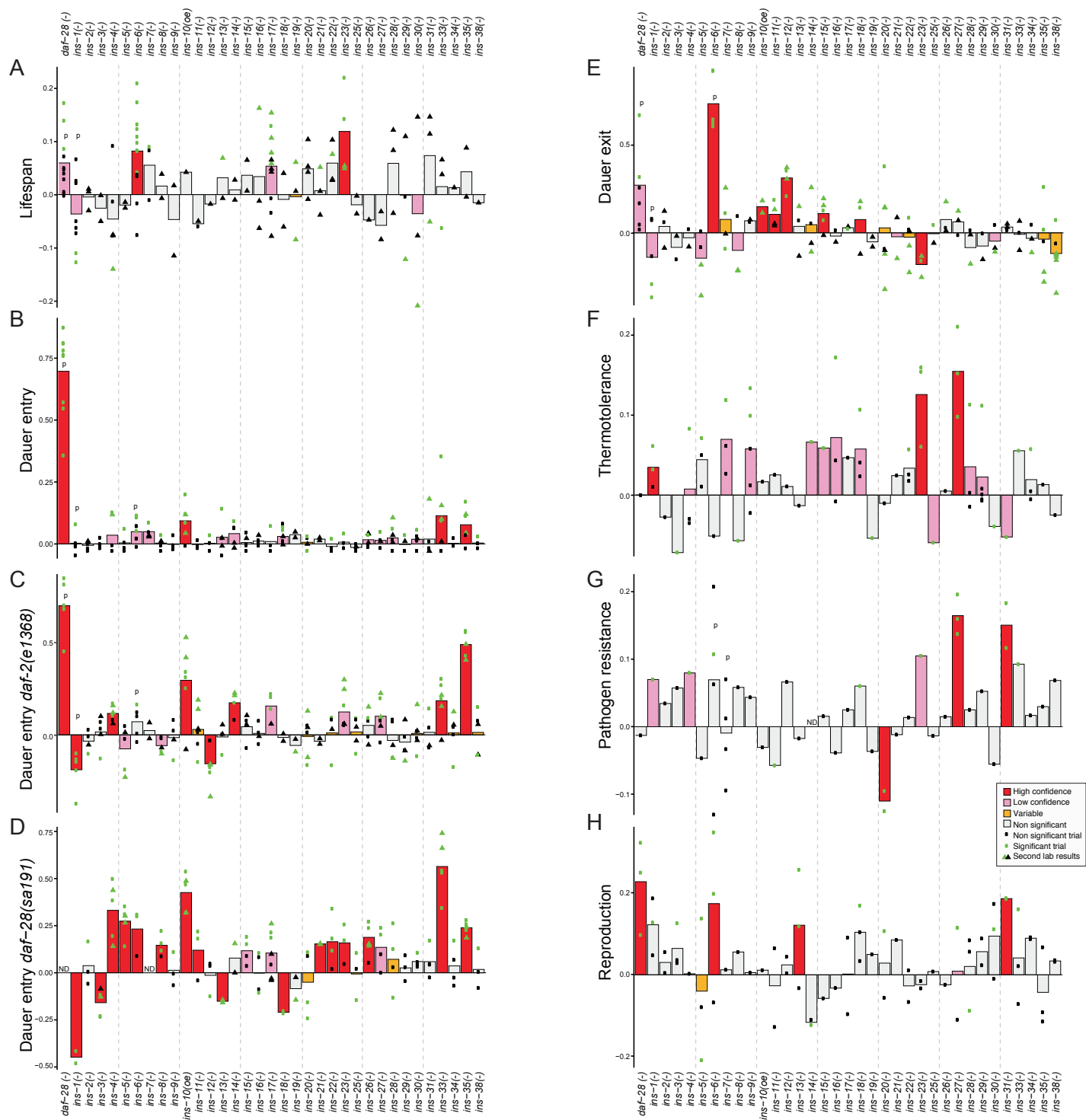

Supplement: Figure S1 — Trial to trial variation in ILP phenotypes. The bar graphs indicate the mean magnitude of each phenotype normalized to same trial controls. The symbols within the bars represent the magnitude for each trial. Phenotypes tested were indicated in the Y-axis: (A) lifespan, (B) dauer entry, (C) dauer entry in the daf-2(e1368) background, (D) dauer entry in the daf-28(sa191) background, (E) dauer exit, (F) thermotolerance, (G) pathogen resistance, and (H) reproductive span. Bars and symbols were colored as indicated in the legend (bottom right). (PDF) [file pgen.1004225.s001.pdf]

Supplemental Figure S2

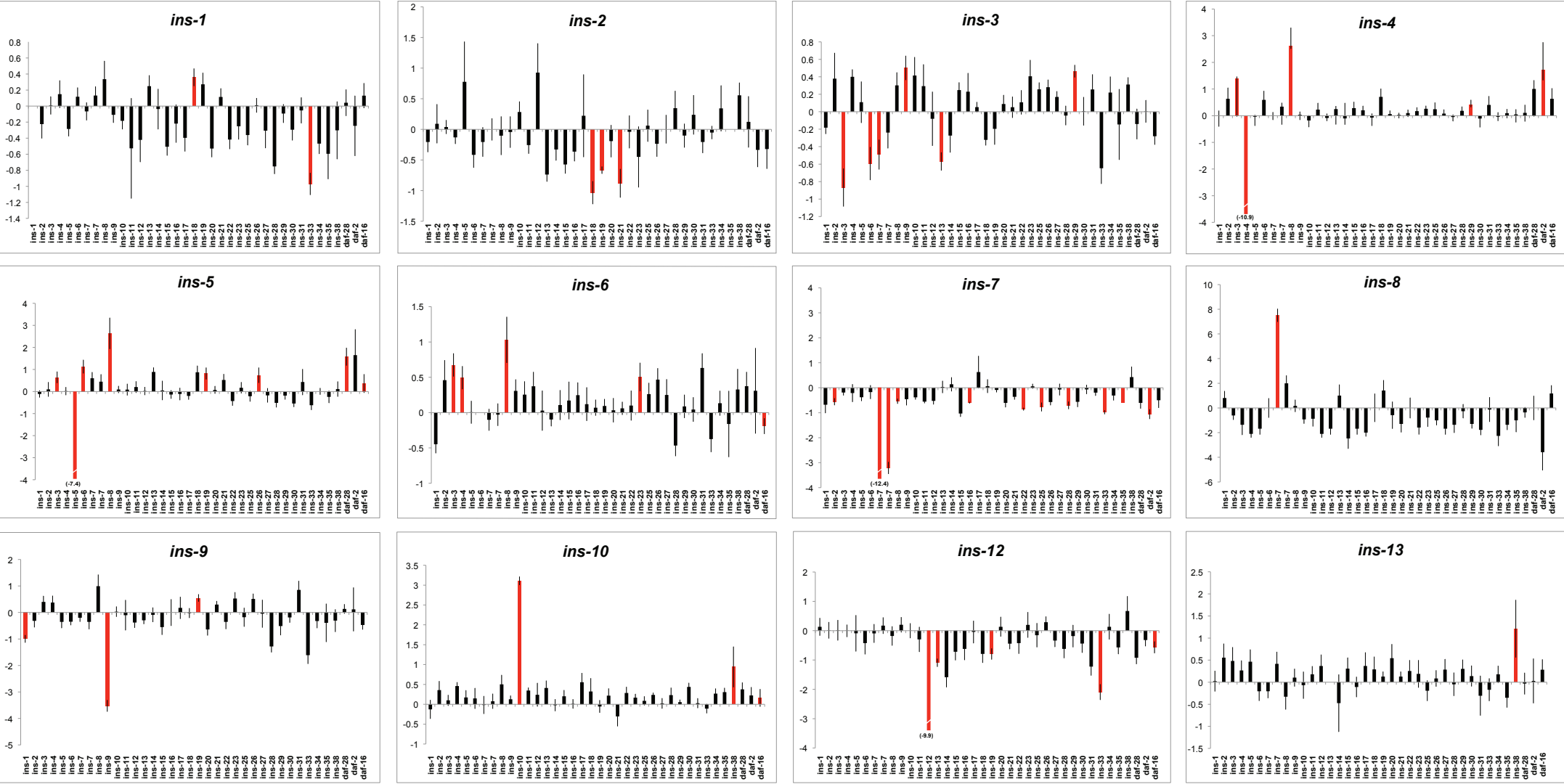

Supplemental Figure S2

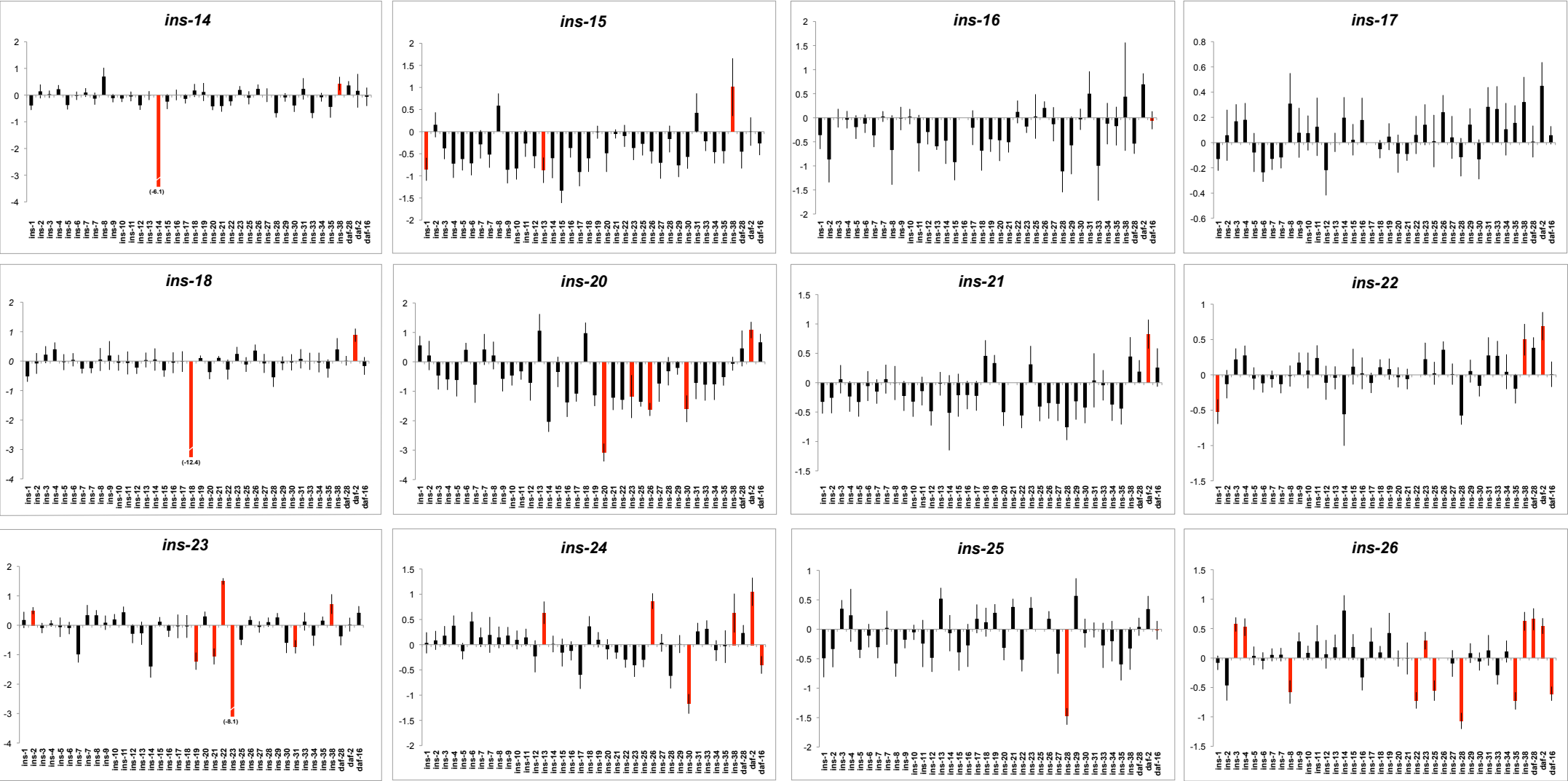

Supplemental Figure S2

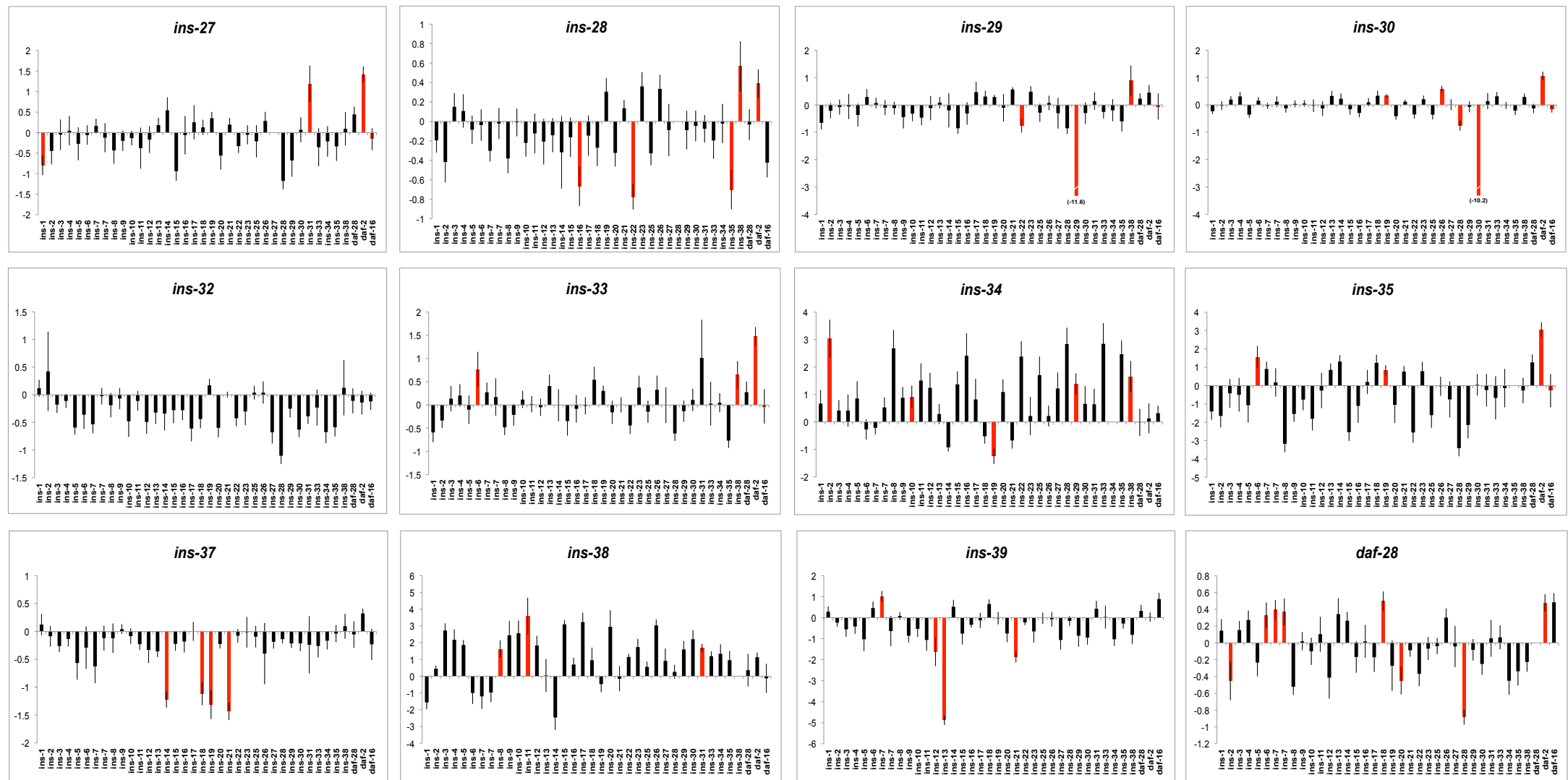

Supplement: Figure S2 — qPCR expression graphs. The graphs represent the fold difference expressions compared to the wild type. Statistical significant changes are highlighted in red. (PDF) [file pgen.1004225.s002.pdf]

Supplemental Figure S3

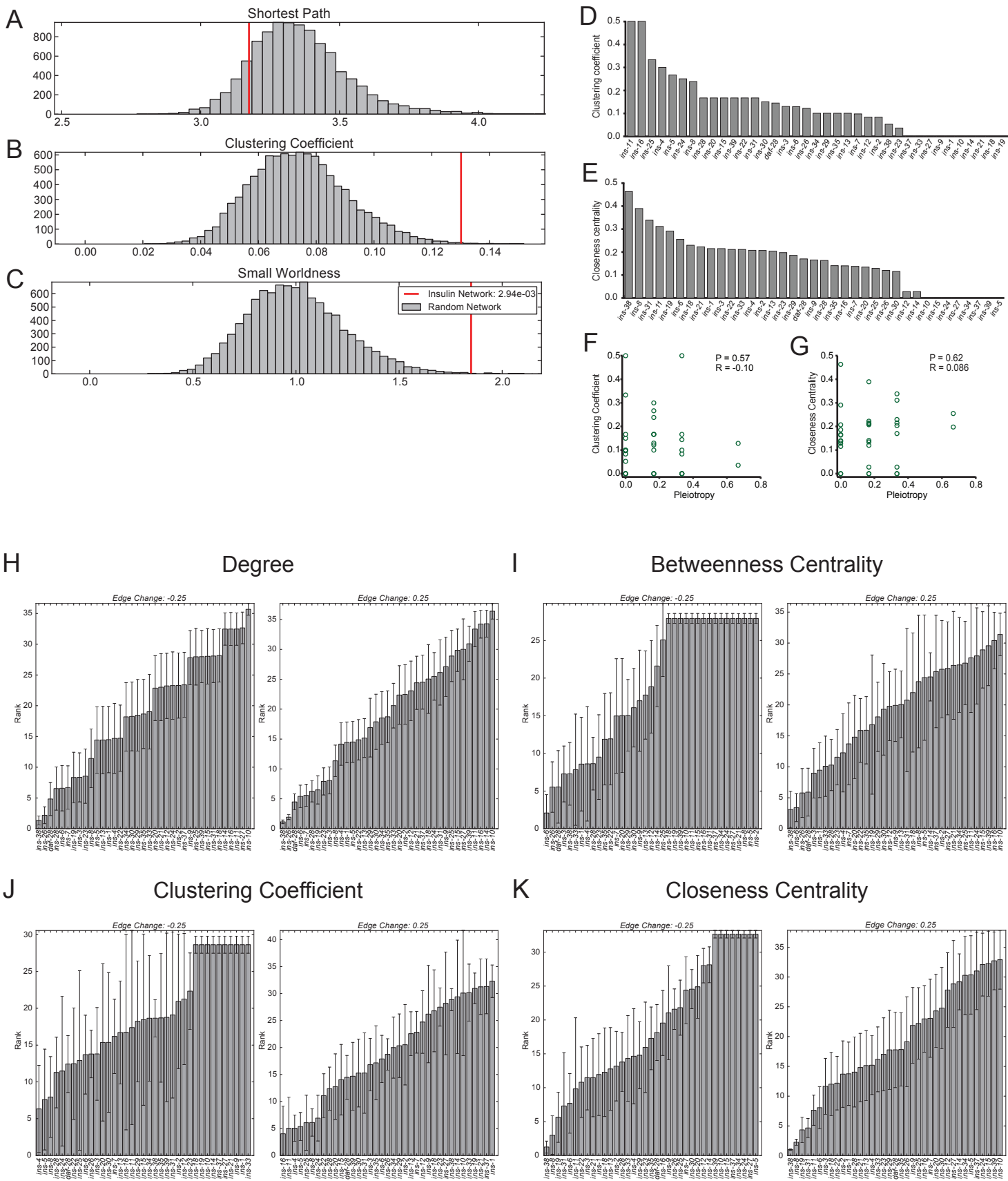

Supplement: Figure S3 — Network robustness and small world properties. Distributions obtained by bootstrapping analysis for (A) shortest path length, (B) clustering coefficient, and (C) small worldness based on the shortest path length and clustering coefficient. The values for the ILP network are indicated by a red vertical line in (A–C). Percentile of the ILP network compared to the random distribution is indicated for small worldness. (D–E) Rank order of the ILPs for clustering coefficient (D) and closeness (E). (F–G) Scatterplot of clustering coefficient (F) and closeness (G) versus pleiotropy as defined in Figure 2 along with P and R values for linear correlation. (H–K) The mean and standard deviation of the ranking after robustness analysis of each gene according to (H) degree, (I) betweenness centrality, (J) clustering coefficient, and (K) closeness centrality. (PDF) [file pgen.1004225.s003.pdf]
